# Comparison of Clinicopathological Characteristics in the Patients with Cardiac Cancer with or without Esophagogastric Junctional Invasion: A Single-Center Retrospective Cohort Study

**DOI:** 10.1155/2013/189459

**Published:** 2013-01-10

**Authors:** Hiroaki Ito, Haruhiro Inoue, Noriko Odaka, Hitoshi Satodate, Michitaka Suzuki, Shumpei Mukai, Yusuke Takehara, Tomokatsu Omoto, Shin-ei Kudo

**Affiliations:** Digestive Disease Center, Showa University Northern Yokohama Hospital, 35-1 Chigasakichuo, Tsuzuki-ku, Yokohama 224-8503, Japan

## Abstract

*Background*. This study addresses clinicopathological differences between patients with gastric cardia and subcardial cancer with and without esophagogastric junctional invasion. *Methods*. We performed a single-center, retrospective cohort study. We studied patients who underwent curative surgery for gastric cardia and subcardial cancers. Tumors centered in the proximal 5 cm of the stomach were classed into two types, according to whether they did (Ge) or did not (G) invade the esophagogastric junction. *Results*. A total of 80 patients were studied; 19 (73.1%) of 26 Ge tumors and 16 (29.6%) of 54 G tumors had lymph nodes metastases. Incidence of nodal metastasis in pT1 tumors was significantly higher in the Ge tumor group. No nodal metastasis in cervical lymph nodes was recognized. Only two patients with Ge tumors had mediastinal lymph node metastases. Incidence of perigastric lymph node metastasis was significantly higher in those with Ge tumors. Ge tumors tended to be staged as progressive disease using the esophageal cancer staging manual rather than the gastric cancer staging manual. *Conclusion*. Because there are some differences in clinicopathological characteristics, it is thought to be adequate to distinguish type Ge from type G tumor.

## 1. Background

Gastric cancer is the fourth most common cancer in the world, and the second most common cause of cancer-related death, affecting approximately 736,000 people in 2008 [1]. Gastric cardia cancer is reportedly increasing in Western countries [2, 3]. Gastric cancers with epicenters in the proximal stomach within 5 cm from esophagogastric junction (EGJ) and crossing EGJ are categorized as esophageal cancers by the American Joint Committee on Cancer/International Union Against Cancer (AJCC/UICC) Cancer Staging Manual [4, 5]. AJCC/UICC also categorizes any gastric cardia cancer without EGJ invasion as gastric cancer regardless of its location. On the other hand, Siewert's classification is widely used as classification for EGJ cancer (EGJC) [6, 7]. Siewert's classification defines gastric cardia adenocarcinoma with its epicenter in the proximal 2 cm of the stomach as type II; subcardial gastric adenocarcinoma with its epicenter in the proximal 5 cm of the stomach, which infiltrates the EGJ and distal esophagus, as type III. Thus, adenocarcinomas with epicenters in the proximal 5 cm of the stomach were defined as type II or III if they infiltrated the EGJ. Siewert et al. also advised that subtotal esophagectomy had less survival effectiveness for patients with type II adenocarcinoma [8]. Therefore, EGJ invasion is an important factor in diagnosis and treatment for gastric cardia cancer. 

The aim of this study was to clarify the differences in clinicopathological characteristics in patients with gastric cardia cancer with or without EGJ invasion and to investigate optimal clinical management of gastric cardia cancer.

## 2. Methods

### 2.1. Study Design

We retrospectively studied patients who underwent curative surgery, including lymph node dissection, for gastric cardia and subcardial cancers at the Digestive Disease Center, Showa University Northern Yokohama Hospital between October 2001 and December 2010. Clinical and histological data and prognoses were determined based on medical records. 

### 2.2. Patients

Patients who underwent curative surgery for gastric cardia or subcardial cancer were included in this study. Inclusion criteria were (i) presence of histologically proven carcinoma of the upper-third of the stomach; (ii) histologically solitary tumors; (iii) no prior treatment by endoscopic resection, chemotherapy, or radiation therapy. Exclusion criteria were (i) presence of synchronous or metachronous malignancy; (ii) presence of severe organ dysfunction. All disease was pathologically staged using the AJCC/UICC TNM Cancer Staging Manual (7th ed.). All patient data were approved for use by the institutional review board of Showa University Northern Yokohama Hospital. This study was registered with the University Hospital Medical Information Network in Japan, number UMIN000008774. 

### 2.3. Classification

Any gastric cancer entered in the proximal 5 cm of the stomach with EGJ invasion is defined as esophageal cancer by the AJCC/UICC, whereas any cancer near the EGJ without EGJ invasion is defined as gastric cancer. Thus, we categorized tumors centered in the proximal 5 cm of the stomach into two types, according to presence of EGJ invasion. Categorization criteria were (i) any histological carcinoma centered in the stomach within 5 cm from EGJ, with EGJ invasion (type Ge); (ii) any histological carcinoma centered in the stomach within 5 cm from EGJ, without EGJ invasion (type G). Type Ge tumors were staged using both esophageal cancer and gastric cancer staging manuals. 

### 2.4. Statistical Analysis

Statistical analysis was performed using JMP Statistical Discovery 9.0.3 (SAS Institute, Cary, USA). Fisher's exact test and *χ* ^2^ test were used to compare characteristics of patients. The nonparametric Mann-Whitney test was used to assess differences in age and tumor size. Kaplan-Meier curves of estimated overall survival were generated and compared between the groups using a 2-sided log-rank test. To investigate prognostic factors, Cox proportional hazard analysis was used. *P* values <0.05 were considered statistically significant.

## 3. Results

### 3.1. Clinicopathological Characteristics

A total of 80 patients were eligible and studied. Median follow-up period of the surviving patients was 32.5 months. Clinicopathological characteristics of the patients are summarized in Table 1. Tumor characteristics were described using AJCC/UICC TNM classifications. Approximately 76% of patients were men; their average age was 67.6 years (range: 35–90 years). Thirty-one (38.8%) and 49 (61.3%) patients underwent proximal gastrectomies and total gastrectomies, respectively. Histologically, all 80 tumors were adenocarcinoma. Twenty-six of 80 patients had tumors with esophageal invasion (type Ge tumor). The mean number of dissected lymph nodes was 38.3 ± 29.9 (SD) in each patient. Thirty-five (43.8%) of the 80 patients had lymph node metastases (pN1–3). Thirty-two (40.0%), 21 (26.3%), 12 (15.0%), and 15 (18.8%) patients were pathologically staged I, II, III, and IV, respectively. 

Comparison of clinicopathological characteristics between type Ge and G tumor groups are summarized in Table 2. There were significant differences in pathological tumor size, distance between EGJ and tumor center, lymphatic invasion, venous invasion, depth of tumor invasion (pT category), lymph node metastasis (pN category), distant metastasis (pM category) and TNM stage. Patients with Type G tumors tended to have earlier-stage diseases. 

Incidence of lymph node metastases is summarized in Table 3. Nineteen (73.1%) of 26 type Ge tumors and 16 (29.6%) of 54 type G tumors had lymph nodes metastases (*P* < 0.001). Although incidence of nodal metastasis in pT1 tumors was significantly higher in the type Ge tumor group than the type G tumor groups, there was no significant difference in pT2, pT3, and pT4 tumors between two tumor groups. No nodal metastases in the cervical lymph node were seen in the type Ge and G tumor groups. Only two patients in type Ge tumor group had mediastinal lymph node metastases (lower thoracic and esophageal hiatal paraesophageal lymph node). Two patients with mediastinal lymph node metastases died. One patient had disease recurrence (peritoneal dissemination), and the other patient died from surgical complication (pneumonia). Incidence of perigastric lymph node metastasis were significantly higher in the type Ge tumor group than in the type G tumor group (*P* = 0.002). In perigastric lymph nodes, the type Ge tumor group had a significantly higher incidence of nodal metastasis at left paracardial (*P* = 0.004), right paracardial (*P* = 0.006), and lesser curvature (*P* = 0.028) lymph nodes. Suprapyloric and infrapyloric lymph nodes metastases were rare in both tumor groups. Type Ge tumor group also had significantly higher incidence of nodal metastasis at the splenic hilum (*P* = 0.032).

### 3.2. Staging Controversy in Gastric Cardia and Subcardial Cancers That Cross the Esophagogastric Junction

**Figure 1:**
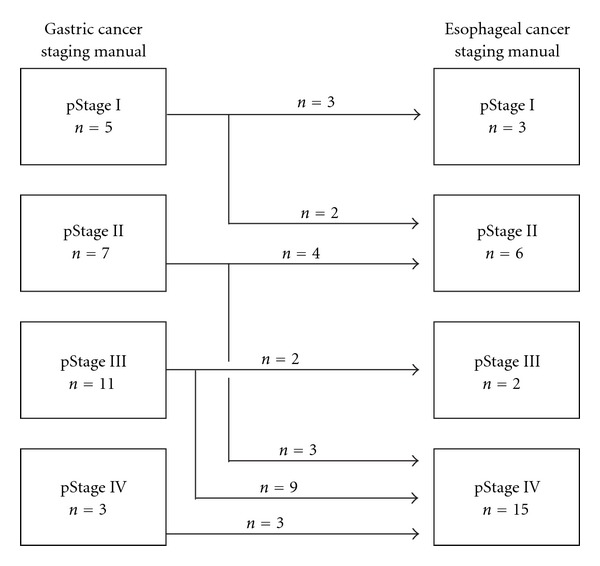
Staging comparison of gastric cardia cancer with esophageal invasion (*n* = 26).

Although the usual tumor location of type Ge tumors is in the stomach, AJCC/UICC Cancer Staging Manual defines type Ge tumors as esophageal cancer. We investigated discrepancies between disease stages by the AJCC/UICC TNM gastric cancer and esophageal cancer staging manual (7th ed.). The stage discrepancy was visualized in Figure 1. Type Ge tumors tended to be staged into progressive disease by esophageal cancer staging manual. Two of 5 in pStage I, 3 of 7 pStage II, and 9 of 11 pStage III patients staged by gastric cancer staging manual were changed more progressive disease by esophageal cancer staging manual. 

### 3.3. Surgical Outcomes

**Figure 2:**
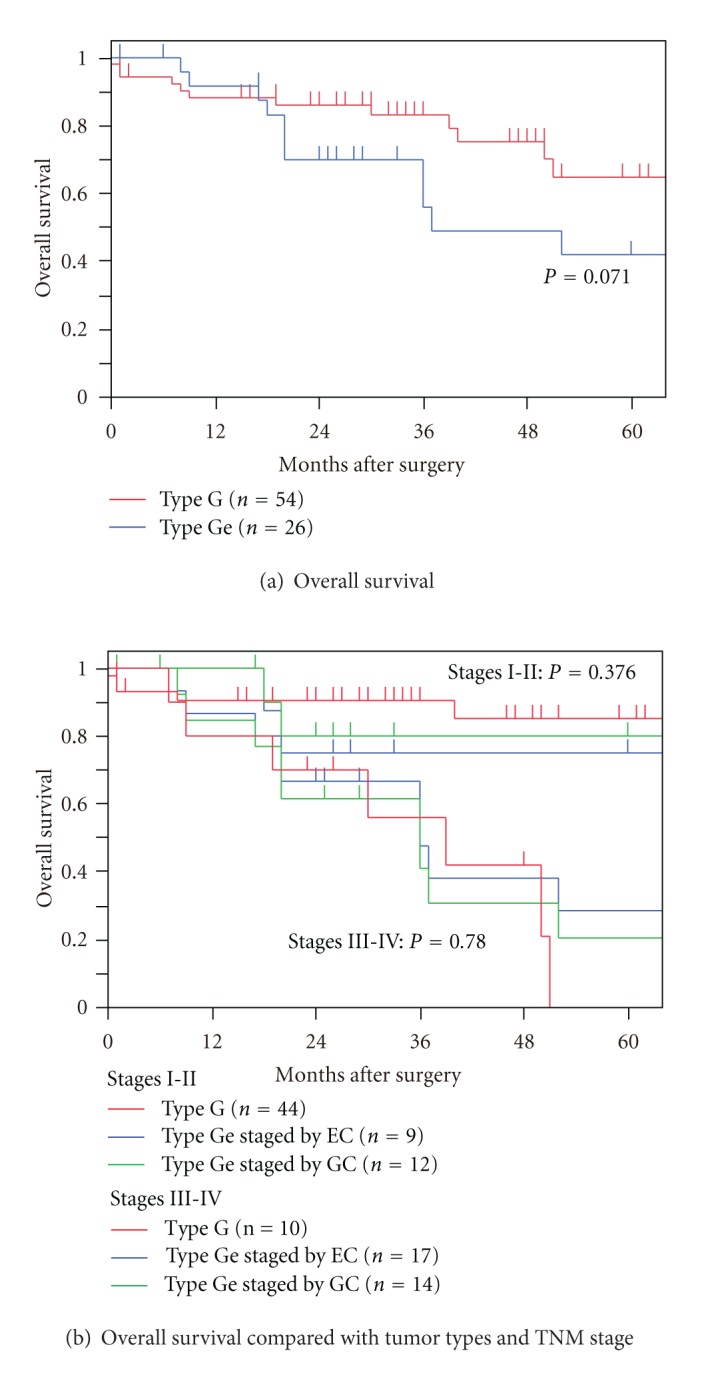
(a) Overall survival rates were compared between the patients with type G and Ge tumors. In patients with pT1–4 tumors, although not significantly, the type G tumor group had a higher survival rate than the type Ge tumor group (*P* = 0.071). (b) Type Ge tumor group was staged by both esophageal and gastric cancer staging manuals. In stages I-II patients, type G tumor group had higher survival rate; however, there was no significance (*P* = 0.376). Although there was no significant difference, survival rate of type Ge tumor group staged by gastric cancer staging manual was superior to those staged by esophageal cancer staging manual. In stages III-IV patients, three patient groups (type G tumor group, type Ge tumor group staged by esophageal cancer staging manual and type Ge tumor group staged by gastric cancer staging manual) had similar survival curves (*P* = 0.780).

The 5-year overall survival rate was 57.3%. Twenty-five patients had recurrent diseases (peritoneum: 11; lymph nodes: 9; liver: 3; lung: 3; bone: 1; adrenal gland: 1; anastomosis: 1), and 24 patients died. Eighteen, 1, and 5 of 24 patients died of cancer, surgical complication, and other causes. Overall survival rates were compared between the patients with type G and Ge tumors. In patients with pT1–4 tumors, although not significantly, the type G tumor group had a higher survival rate (5-year overall survival rate, 64.9%) than the type Ge tumor group (5-year overall survival rate, 49.0%) (*P* = 0.071; Figure 2(a)). Next, type Ge tumor group was staged by both esophageal and gastric cancer staging manuals. In stages I-II patients, type G tumor group had higher survival rate; however; there was no significance (*P* = 0.376). Although there was no significant difference, survival rate of type Ge tumor group staged by gastric cancer staging manual was superior to those staged by esophageal cancer staging manual. In stage III-IV patients, three patient groups (type G tumor group, type Ge tumor group staged by esophageal cancer staging manual, and type Ge tumor group staged by gastric cancer staging manual) had similar survival curves (*P* = 0.780) (Figure 2(b)). 

## 4. Discussion

The aim of this study was to clarify the clinicopathological differences between gastric cardia cancer with and without esophageal invasion. Gastric cardia cancers with esophageal and EGJ invasion have been treated as EGJC [9] or gastric [10] cancer. However, AJCC/UICC TNM Cancer Staging Manual (7th ed.) defined tumors centered in the proximal 5 cm of the stomach that cross the EGJ as esophageal cancer [4, 5]; therefore, we staged those using the esophageal cancer staging manual. 

Siewert et al. argued that complete surgical resection and lymph node metastasis were independent prognostic factors in EGJC, and subtotal esophagectomy had less survival effectiveness for the patients with type II adenocarcinoma [8]. Hasegawa et al. reported that about 60% and 90% of patients with type II and III tumors, respectively, had lymph node metastases and recommended complete resection for improving survival [11]. Schiesser et al. reported that extended total gastrectomy should be performed for type II-III tumors [12]. With regard to surgical approach, Sasako et al. showed that the left thoracoabdominal approach did not improve survival after the abdominal-transhiatal approach and leads to increased morbidity in patients with cancer of the cardia or subcardia [13]. Carboni et al. maintained effects of extended gastrectomy by an abdominal-transhiatal approach for EGJC [14]. 

We studied any tumor centered in the proximal 5 cm of the stomach, and simply categorized them in 2 groups including types Ge and G according to the presence of EGJ invasion. Whereas there were significantly differences in patients background between type Ge and G tumor groups, type G tumor group showed some differences in clinicopathological characteristics. In lymph node metastasis, approximately 70% and 30% of the patients with type Ge and G tumors, respectively, had lymph node metastases in this study. Although no cervical lymph node metastasis was recognized in either tumor group, lower mediastinal lymph node metastasis was recognized in only the type Ge tumor group. Especially, there was significant difference of incidence of nodal metastasis in left paracardial, right paracardial, and lesser curvature lymph node between two tumor groups. To achieve complete resection, we should perform partial or total gastrectomy and lower esophagectomy with lower mediastinal and abdominal lymphadenectomy for the type Ge tumor. For type G tumor, it was not necessary to perform mediastinal lymphadenectomy because there was no mediastinal lymph node metastasis in the type G tumor group.

Because EGJ invasion generally occurs with tumor progression in gastric cancer, it is reasonable that type Ge tumors have deeper depth of invasion and more widespread nodal metastasis than type G tumors. Additionally, esophageal invasion could increase risk of mediastinal lymph node metastasis. Therefore, the survival rate of patients with type Ge tumor was inferior, but not significantly, to patients with type G tumor. 

The AJCC/UICC TNM staging system for esophageal cancer defines nodal metastasis at perigastric lymph nodes (other than the left and right paracardial lymph nodes) as distant metastasis [4, 5], although the lymph node along the lesser curvature is a key regional lymph node for gastric cancer. Thus, type Ge tumors tended to be staged into progressive disease by the esophageal cancer staging manual. Interestingly, the survival rate of stage I-II patients staged by the esophageal cancer staging manual was inferior to those staged by the gastric cancer staging manual, whereas the survival rate of stages III-IV patients staged by the esophageal cancer staging manual was similar to those staged by the gastric cancer staging manual. However, as the survival rates of type Ge and G tumor groups did not significantly differ, there is no significant evidence for or against using the AJCC/UICC staging manual for these tumors. 

As clinicopathological characteristics and survival between type Ge and G tumor groups differ, it is appropriate to distinguish type Ge with type G tumor; however, it is unclear from this study that we should treat type Ge tumors as esophageal cancer, because this study analyzed too small number of the patients to clarify the validity. 

## 5. Conclusions

Because the AJCC/UICC Cancer Staging Manual categorizes gastric cardia cancer with EGJ invasion as esophageal cancer, EGJ invasion is an important factor in diagnosis and treatment for gastric cardia cancer. We retrospectively studied patients who underwent curative surgery for gastric cardia and subcardial cancers to clarify the differences in clinicopathological characteristics and prognoses in patients with gastric cardia cancer with (type Ge) or without (type G) EGJ invasion. Among patients with pT1 tumor, those with type Ge tumor had significantly higher incidence of lymph node metastasis, whereas mediastinal lymph node metastases were seen in only 2 patients with type Ge tumors.

As patients with Ge tumors staged by either esophageal or gastric cancer staging manuals showed no significant difference in survival rates, it is adequate to distinguish type Ge from type G tumor because of their clinicopathological differences. Therefore, it seems that to regard type Ge tumor as esophageal cancer is adequate. 

## Figures and Tables

**Table 1:** Clinicopathological characteristics of patients (*n* = 80).

Variable	Number of subject
Age (year, mean ± SD)	67.6 ± 10.8
Sex	
Male	61 (76.3%)
Female	19 (23.7%)
Pathological tumor size (mm, mean ± SD)	46.8 ± 22.7
Macro type	
Type 0	31 (38.8%)
Type 1	4 (5.0%)
Type 2	18 (22.5%)
Type 3	22 (27.5%)
Type 4	1 (1.3%)
Type 5	4 (5.0%)
Main histological type^†^	
Differentiated	48 (60.0%)
Undifferentiated	32 (40.0%)
Distance between EGJ and tumor epicenter	
≤20 mm	21 (26.3%)
>20 mm	59 (73.8%)
Esophageal invasion	
Yes	26 (32.5%)
No	54 (67.5%)
Lymphatic invasion	
L0	32 (40.0%)
L1	48 (60.0%)
Venous invasion	
V0	32 (40.0%)
V1	48 (60.0%)
V2	0
Depth of tumor invasion	
pT1	29 (36.3%)
pT2	11 (13.8%)
pT3	26 (32.5%)
pT4	14 (17.5%)
Lymph node metastasis	
pN0	45 (56.3%)
pN1	12 (15.0%)
pN2	11 (13.8%)
pN3	12 (15.0%)
Distant metastasis	
M0	65 (81.3%)
M1	15 (18.8%)
TNM stage	
I	32 (40.0%)
II	21 (26.3%)
III	12 (15.0%)
IV	15 (18.8%)
Extent of gastrectomy	
Proximal	31 (38.8%)
Total	49 (61.3%)
Surgical approach	
Laparoscopic surgery	30 (37.5%)
Hand-assisted laparoscopic surgery	17 (21.3%)
Open surgery	33 (41.3%)
Splenectomy	
No	56 (70.0%)
Yes	24 (30.0%)
Thoracotomy	
No	74 (92.5%)
Yes	6 (7.5%)
Postoperative chemotherapy	
No	45 (56.3%)
Yes	35 (43.8%)

^†^Differentiated: papillary carcinoma, well differentiated adenocarcinoma, moderately differentiated adenocarcinoma; Undifferentiated: poorly differentiated adenocarcinoma, signet-ring cell carcinoma, mucinous adenocarcinoma.

**Table 2:** Comparison of clinicopathological characteristics of patients with gastric cardia cancer with or without esophageal invasion.

Variable	With esophageal invasion (*n* = 26)	Without esophageal invasion (*n* = 54)	*P*-value
Age (year, mean ± SD)	65.3 ± 10.8	68.6 ± 10.8	0.271
Sex			0.773
Male	19 (73.1%)	42 (77.8%)	
Female	7 (26.9%)	12 (22.2%)	
Pathological tumor size (mm, mean ± SD)	61.9 ± 18.9	39.5 ± 20.8	<0.001**
Main histological type^†^			0.811
Differentiated	15 (57.7%)	33 (61.1%)	
Undifferentiated	11 (42.3%)	21 (38.9%)	
Distance between EGJ and tumor epicenter			<0.001**
≤20 mm	15 (57.7%)	6 (11.1%)	
>20 mm	11 (42.3%)	48 (88.9%)	
Lymphatic invasion			0.003**
L0	4 (15.4%)	28 (51.9%)	
L1	22 (84.6%)	26 (48.2%)	
Venous invasion			<0.001**
V0	3 (11.5%)	29 (53.7%)	
V1	23 (88.5%)	25 (46.3%)	
Depth of tumor invasion			0.028*
pT1	4 (15.4%)	25 (46.3%)	
pT2	3 (11.5%)	8 (14.8%)	
pT3	13 (50.0%)	13 (24.1%)	
pT4	6 (23.1%)	8 (14.8%)	
Lymph node metastasis			0.002**
pN0	7 (26.9%)	38 (70.4%)	
pN1	6 (23.1%)	6 (11.1%)	
pN2	5 (19.2%)	6 (11.1%)	
pN3	8 (30.8%)	4 (7.4%)	
Distant metastasis			0.010*
M0	11 (42.3%)	54 (100%)	
M1	15 (57.7%)	0	
TNM stage			<0.001**
I	3 (11.5%)	29 (53.7%)	
II	6 (23.1%)	15 (27.8%)	
III	2 (7.7%)	10 (18.5%)	
IV	15 (57.7%)	0	
Extent of gastrectomy			0.150
Proximal	7 (26.9%)	24 (44.4%)	
Total	19 (73.1%)	30 (55.6%)	
Surgical approach			0.037*
Laparoscopic surgery	6 (23.1%)	24 (44.4%)	
Hand-assisted laparoscopic surgery	4 (15.4%)	13 (24.1%)	
Open surgery	16 (61.5%)	17 (31.5%)	
Splenectomy			<0.001**
No	9 (34.6%)	47 (87.0%)	
Yes	17 (65.4%)	7 (13.0%)	
Thoracotomy			0.013*
No	21 (80.8%)	53 (98.2%)	
Yes	5 (19.2%)	1 (1.9%)	
Postoperative chemotherapy			0.002**
No	8 (30.8%)	37 (68.5%)	
Yes	18 (69.2%)	17 (31.5%)	

*P < 0.05, **P < 0.01.

^†^Differentiated: papillary carcinoma, well differentiated adenocarcinoma, moderately differentiated adenocarcinoma; Undifferentiated: poorly differentiated adenocarcinoma, signet-ring cell carcinoma, mucinous adenocarcinoma.

**Table 3:** Comparison of lymph node metastasis in patients with gastric cardia cancer, with or without esophageal invasion.

Variable	With esophageal invasion (*n* = 26)	Without esophageal invasion (*n* = 54)	*P*-value
Overall	19 (73.1%)	16 (29.6%)	<0.001∗∗
Depth of tumor invasion			
pT1	2/4 (50.0%)	0/15	0.035*
pT2	2/3 (66.7%)	4/8 (50.0%)	0.576
pT3	9/13 (69.2%)	7/13 (53.8%)	0.344
pT4	6/6 (100%)	5/8 (62.5%)	0.154
Location of lymph node			
Cervical LN	0	0	—
Mediastinal LN	2 (7.7%)	0	0.103
Perigastric LN	17 (65.4%)	15 (27.8%)	0.002**
Left paracardial	8 (30.8%)	3 (5.6%)	0.004**
Right paracardial	10 (38.5%)	6 (11.1%)	0.006**
Lesser curvature	12 (46.2%)	12 (22.2%)	0.028*
Greater curvature	4 (15.4%)	2 (3.7%)	0.100
Suprapyloric	0	1 (1.9%)	0.675
Infrapyloric	1 (3.8%)	0	0.325
Left gastric artery	4 (15.4%)	7 (13.0%)	0.508
LN at Celiac trunk	1 (3.8%)	2 (3.7%)	0.698
LN along hepatic artery	2 (7.7%)	1 (1.9%)	0.245
LN along splenic artery	3 (11.5%)	1 (1.9%)	0.098
LN at splenic hilum	3 (11.5%)	0	0.032*

*P < 0.05, **P < 0.01.
